# Plasmacytoid dendritic cells: development, functions, and role in atherosclerotic inflammation

**DOI:** 10.3389/fphys.2014.00279

**Published:** 2014-07-25

**Authors:** Dimitry A. Chistiakov, Alexander N. Orekhov, Igor A. Sobenin, Yuri V. Bobryshev

**Affiliations:** ^1^Department of Medical Nanobiotechnology, Pirogov Russian State Medical UniversityMoscow, Russia; ^2^Laboratory of Angiopathology, Institute of General Pathology and Pathophysiology, Russian Academy of Medical SciencesMoscow, Russia; ^3^Institute for Atherosclerosis Research, Skolkovo Innovative CenterMoscow, Russia; ^4^Laboratory of Medical Genetics, Russian Cardiology Research and Production ComplexMoscow, Russia; ^5^Faculty of Medicine, University of New South WalesSydney, NSW, Australia; ^6^School of Medicine, University of Western SydneyCampbelltown, NSW, Australia

**Keywords:** plasmacytoid dendritic cell, conventional dendritic cell, innate immunity, type I interferon, inflammation, tolerance, atherosclerosis

## Abstract

Plasmacytoid dendritic cells (pDCs) are a specialized subset of DCs that links innate and adaptive immunity. They sense viral and bacterial pathogens and release high levels of Type I interferons (IFN-I) in response to infection. pDCs were shown to contribute to inflammatory responses in the steady state and in pathology. In atherosclerosis, pDCs are involved in priming vascular inflammation and atherogenesis through production of IFN-I and chemokines that attract inflammatory cells to inflamed sites. pDCs also contribute to the proinflammatory activation of effector T cells, cytotoxic T cells, and conventional DCs. However, tolerogenic populations of pDCs are found that suppress atherosclerosis-associated inflammation through down-regulation of function and proliferation of proinflammatory T cell subsets and induction of regulatory T cells with potent immunomodulatory properties. Notably, atheroprotective tolerogenic DCs could be induced by certain self-antigens or bacterial antigens that suggests for great therapeutic potential of these DCs for development of DC-based anti-atherogenic vaccines.

## Introduction

Atherosclerotic arterial disease is a chronic cardiovascular pathology commonly associated with heart attack and stroke, both are leading causes of mortality in developed countries. Various cardiometabolic risk factors including biased plasma levels of lipoproteins could induce endothelial dysfunction progressing to preclinical atherogenesis even early in life (Morrison et al., 2013). Entrance and accumulation of plasma lipids (and modified lipoproteins especially) in the arterial wall launches the adaptive immune response. Blood-borne immune cells infiltrate intimal regions enriched with lipids. Although macrophages and some dendritic cells (DCs) reside in the arterial wall, increased influx of extravasated monocytes is thought to be the major trigger of inducing the inflammatory response in affected intima media (Ley et al., 2011). In the intima media, monocytes acquire a phenotype that is consistent with inflammatory macrophages and inflammatory DCs and is influenced by load of modified lipids, cytokines, chemokines, and hematopoietic growth factors. Due to the proinflammatory switch in the local microenvironment, resident macrophages and DCs also change their phenotype (Lech et al., 2012).

Along with monocytes and macrophages, DCs play a key role in early stages of atherosclerotic inflammation and in advanced stages of atherosclerosis (Bobryshev and Lord, 1998; Bobryshev, 2010). DCs are professional antigen-presenting cells (APCs) involved in the induction of T cell-mediated adaptive immunity through the recruitment of naïve T cells. However, in atherogenic proinflammatory conditions, the normal adaptive immune response becomes maladaptive. A variety of DC subsets is present in lymphoid and non-lymphoid organs. Two major DC subpopulations include conventional DCs (cDCs) and plasmacytoid DCs (pDCs) (Miloud et al., 2010). During inflammation, an additional DC subset has been described, so-called inflammatory DCs, which differentiate from monocytes recruited to the site of inflammation (Segura and Amigorena, 2013). In this review, we highlight the recent information about the development and functions of pDCs as well as role of this subtype of DCs in atherosclerotic inflammation.

## Development of pDCs

The classical hematopoietic model states the segregation of lymphoid and myeloid lineages at earliest stages of hematopoiesis. In early studies, murine cDCs and pDCs were suggested to develop through either the myeloid or the lymphoid pathway of hematopoiesis (Diao et al., 2004). However, accumulated evidence indicates that the origin of human DCs is markedly different from that of murine DCs. An existence of a common DC precursor was suggested for human cDCs and pDCs (Ishikawa et al., 2007). In mice, Naik et al. (2007) then reported finding unique CD11c^−^MHCII^−^ DC progenitors that differentiate to CD11c^+^MHCII- DC precursors further generating the three distinct CD11c^+^MHCII^+^ DC subtypes (pDCs, CD8^+^ cDCs, and CD8^−^ cDCs). Both murine DC progenitors were shown to express surface fms-like tyrosine kinase 3 (Flt3), a receptor for the hematopoietic growth factor Flt3 ligand (Naik et al., 2010).

**Figure 1:**
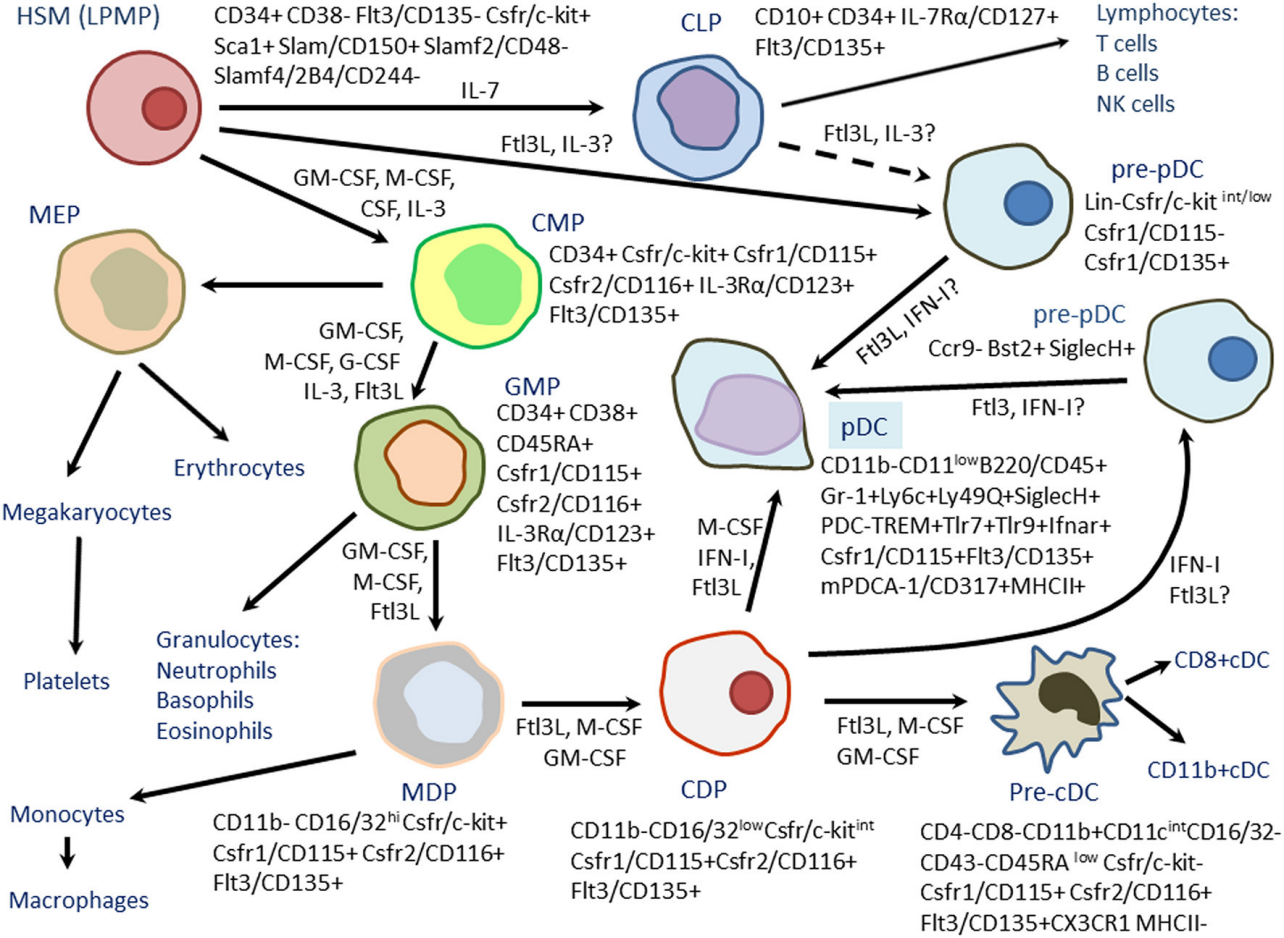
**Differentiation of mouse plasmacytoid dendritic cells (pDCs) from hematopoietic stem cells (HSCs) or lymphoid-primed multipotent progenitors (LPMPs)**. Bone-marrow hematopoiesis is a multistep process involving sequential generation of common lymphoid progenitors (CLPs), common myeloid progenitors (CMPs), megakaryote-erythroid progenitors (MEPs), granulocyte-macrophage progenitors (GMPs), macrophage dendritic cell progenitors (MDPs), common dendroid cell progenitors (CDP), and conventional dendritic cell precursors (pre-cDCs). Generally, MDPs and CDPs preferentially differentiate to cDCs and produce few pDCs. However, two subsets of pDC precursors (Lin^−^c-kit^int/low^CD115^−^CD135^+^ and CCr9^−^Bst2^+^SiglecH^+^) with high potential to differentiate to pDCs were found. These subsets could be generated from either from CDPs or early lymphoid progenitors (LPMPs, CLPs) suggesting for the existence of the alternate mechanisms of pDC differentiation in mice. Phenotypes of hematopoietic progenitors are presented. Growth factors and cytokines stimulating hematopoietic differentiation are shown near arrows.

CD11c^−^MHCII^−^Flt3^+^ DC precursors seem to represent common myeloid progenitors (CMPs) that differentiate to DC-restricted MHCII-Flt3+ DC progenitors expressing colony-stimulating factor 1 receptor (CSF1R) and capable to produce both cDCs and pDCs (Onai et al., 2007) (Figure 1). Macrophage-colony-stimulating factor (M-CSF) was shown to bind to CSF1R and drive differentiation of both cDCs and pDCs in mice (Fancke et al., 2008). DC-restricted MHCII-Flt3+ DC progenitors then differentiate into the common CD11c^+^MHC^−^II^−^ cDC-restricted precursors (called pre-DCs) further producing CD8^+^ and CD11b^+^ cDCs (Diao et al., 2006; Naik et al., 2006). Common lymphoid DC progenitors (CLPs) were found in the mouse bone marrow (D'Amico and Wu, 2003; Sathe et al., 2013). According to the study of Sathe et al. (2013), CLPs produced only a few cDCs with variable efficiency, but produced pDCs *via* a transient intermediate precursor with B-cell potential. pDCs of CLP origin showed evidence of past recombination activating gene (RAG)-1 expression and had D-J IgH gene rearrangements suggesting for their lymphoid past (Corcoran et al., 2003; Pelayo et al., 2005). Differentiation of CMPs resulted in a heterogeneous population of pDCs. Most pDCs of CMP origin did not show signs of a lymphoid past. However, some pDCs of CMP origin exhibited evidence of past RAG1 expression and had D-J IgH gene rearrangements (Sathe et al., 2013). Some CMP-derived pDCs were without such IgH gene rearrangements. Finally, some pDC-like cells did not express key pDC markers such as C-C chemokine receptor (CCR)-9 but produced interferon (IFN)-α, a characteristic of the pDC subset (Shortman et al., 2013). Upon stimulation with CpG oligonucleotides, pDCs of both CLP and CMP origin secreted IFN-α. Indeed, both pDCs and cDCs could be convergently generated from the lymphoid and myeloid precursors.

Like mouse pDCs, human pDCs could be convergently produced from the lymphoid (granulocyte-macrophage) and multi-myeloid progenitors (MLPs) that are distinct from the conventional myeloid or lymphoid pathway (Ishikawa et al., 2007). Consistent with this, mutation in GATA2, a key hematopoietic transcription factor fully abolishes population of human MLPs and results in complete lack of DCs (Collin et al., 2011). Flt3 ligand in synergy with GM-CSF, IL-4, and tumor necrosis factor (TNF)-α was shown to act as a potent inducer of myeloid DC precursors from hematopoietic precursors. Myeloid precursors could be then clonally expanded in the presence of Flt3 ligand, GM-CSF, and thrombopoietin (TPO). Finally, Flt3 ligand supports further maturation of myeloid precursors to functional CD1a^+^ DC precursors (Harada et al., 2007).

However, compared to the knowledge about differentiation of mouse pDCs, the developmental stages of different DC subsets in humans remain poorly defined (Schotte et al., 2010). To date, human equivalents of mouse macrophage dendritic cell progenitor (MDP), common DC progenitor (CDP), and pre-DC have not been found. Compared to mouse DC precursors, human CD34^+^ hematopoietic stem cells (HSCs) express major histocompatibility complex (MHC) class II (Majumdar et al., 2003). This indeed hampers the identification of early DC precursors in human blood. In contrast to human cDCs that could proliferate, pDCs do not proliferate suggesting that human pDCs leave the bone marrow fully differentiated (Randolph et al., 2008). Comparison of genome-wide expression profiles clearly cluster human pDCs with mouse pDCs with sharing a large gene expression program shared between those cells (Robbins et al., 2008). That should be helpful in further finding similarities and differences in the developmental programs of human and mouse pDCs.

Depending on the stimuli, DC progenitors are able to develop DCs with different phenotypes. For example, culturing mouse DC progenitors with granulocyte-macrophage colony-stimulating factor (GM-CSF) and interleukin (IL)-4 resulted in generating inflammatory Mac3^+^CD11b^+^CD24^+^ DCs expressing large amounts of tumor necrosis factor (TNF)-α, IL-10, chemokine (C-C motif) ligand (CCL)-2, and nitric oxide (NO). Unlike GM-CSF/IL-4-induced DCs, Flt3-induced DCs produced no TNF-α, IL-10, or CCL-2. Further investigations showed that GM-CSF/IL-4-induced DCs correspond to TNF-α- and NO-producing proinflammatory DCs while FLt3-induced DCs are equivalents of the steady-state resident DCs (Xu et al., 2007). These finding showed that local microenvironment could play a critical role in driving the terminal phenotype of DCs. Indeed, vascular inflammation presented in atherosclerotic lesions will favor for differentiation of circulating DC progenitors and precursors toward the proinflammatory DCs.

## Transciption and growth factors driving differentiation of pDCs

As mentioned above, common myeloid and lymphoid DC progenitors were found in mice and humans. However, since the DC differentiation program is studied much better in mice than in humans, we will focus on murine transcription and growth factors that are involved in DC development.

MDPs precede differentiation of myeloid progenitors to the mononuclear phagocyte lineage in hematopoiesis (Fogg et al., 2006). MDPs produce spleen macrophages, cDCs resided in the lymphoid and non-lymphoid tissue, and a few pDCs (Merad et al., 2013). MDPs have the following phenotype: Lin^−^Sca1^−^IL-7Rα^−^CD116^−^/32^hi^c-kit^+^CX3CR^+^CD11b^−^CD115^+^CD135^+^ (Fogg et al., 2006). The precursors are negative for stem cell markers Lin and Sca1, both are essential for maintenance and self-renewal of HSCs (Kumar et al., 2008), but still express c-kit (or CD117), another surface marker of HSCs. In addition, MDPs express some lymphoid lineage-specific markers such as CX3CR (receptor for fractalkine CX3CL1 that regulates adhesion and migration of lymphocytes) but lose the receptor for IL-7, a cytokine driving development of B cells. MDPs highly express myeloid lineage-specific markers such as CD16/32 (Fc receptors) and CD115 (M-CSF receptor) and start to express Flt3 (CD135), a DC-specific marker. At that stage, hematopoietic growth factors such as GM-CSF, M-CSF, and Flt3 ligand are essential to regulate commitment of mononuclear phagocytes and DC precursors (Stanley et al., 1997; Karsunky et al., 2003; Fancke et al., 2008). In fact, GM-CSF is a critical factor for general DC development under both steady-state and inflammatory conditions including GM-CSF-mediated activation of key signaling modules such as Jak/Stat, Mapk, Pi3k, and NF-κ B that support cell growth and proliferation (van de Laar et al., 2012).

In an MDP, expression of lineage-specific transcription factors such as nuclear factor, interleukin 3 regulated (Nfil3) and interferon regulatory factor-8 (Irf8) is induced to initiate transcriptional programs regulating differentiation toward mononuclear phagocytes and CD8α^+^ cDCs (Wang and Morse, 2009; Becker et al., 2012; Male et al., 2012). Nfil3 is required for CD8α^+^ cCD development since Nfil3-deficient mice specifically lack CD8α^+^ cCDs but not CD8α^−^ cCDs and pDCs (Kashiwada et al., 2011). Transcription factor PU.1 (spleen focus forming virus proviral integration oncogene; Spi1) is essential for chromatin structure remodeling in the promoter of the Irf8 gene to activate a lineage- and developmental-stage-specific *cis*-enhancer to prevent MDP reprogramming toward the myeloid lineage and induce DC-specific differentiation (Schönheit et al., 2013). In the absence of PU.1, MDP spontaneously differentiate to neutrophils. Irf8 is crucial for the development of CD8α+ cDCs mediated by transcription factors such as DNA-binding protein inhibitor (Id2) and basic leucine zipper transcription factor, ATF-like (Batf3) In the absence of Id2 and Batf3, Irf8 supports preferential differentiation of DC precursors to pDCs (Jaiswal et al., 2013).

After commitment of the mononuclear phagocyte lineage, MDPs differentiate to CDPs that phenotypically are as follows: Lin^−^Sca1^−^IL-7Rα^−^CD16/32^low^c-kit^int^CD11b^−^CD115^+^CD135^+^. Compared to MDPs, CDPs have decreased expression of the myeloid lineage marker CD16/32 but hold expression of both CD115 and CD135 necessary for further differentiation and maintenance of DC-specific lineage properties (Naik et al., 2007; Onai et al., 2007). CDPs have increased expression of Ret3, a surface marker specific for DCs and follicular macrophages (follicular DCs) (Nagasaki et al., 1995). Hematopoietic growth factors (GM-CSF, M-CSF, and Flt3 ligand) are required to support differentiation of MDPs to DCs.

Compared to MDPs, expression of the DC lineage-supporting transcription factors such as Nfil3, Irf8, PU.1, signal transducer and activator of transcription (Stat5b), B-cell lymphoma/leukemia 11A (Bcl11a), and Runt-related transcription factor 2 (Runx2), is up-regulated in CDPs (Murphy, 2013). Bcl11a contributes to DC development through activation of expression of Flt3 in early hematopoietic precursors (Wu et al., 2013). Bcl11a is considered as a pDC-specific marker (Pulford et al., 2006; Marafioti et al., 2008) especially critical for commitment of pDCs since it regulates transcription of E2-2 (also known as transcription factor-4; Tcf4) and other DC differentiation modulators including Id2 and core-binding factor, Runt-domain, α-subunit 2, translocated to, 3 (Cbfa2t3/Mtg16) (Ippolito et al., 2014). In Bcl11a-deficient mice, numbers of pDCs were markedly decreased and development of cDCs was impaired (Wu et al., 2013).

PU.1, an ETS family transcription factor, is required for supporting preferential commitment of common DC precursors from common myeloid precursors (Carotta et al., 2010) and induction of expression of CD11c, a specific surface marker of pDCs in DC precursors (Zhu et al., 2012). However, PU.1 is not a transcription factor that supports predominant differentiation of DC precursors to pDC. In support of this, Schlitzer et al. (2011) showed the ability of CCR9^−^MHCII^low^ pDCs, which are immediate precursors of fully differentiated CCR9^+^ pDCs, to switch maturation to CD11b^+^CD8^−^MHCII^high^ cDCs in the presence of GM-CSF that down-regulates E2-2 and up-regulates PU1.2, Id2, and Batf3.

In Runx2-deficient mice, pDCs developed normally in the bone marrow but were greatly reduced in the periphery suggesting that Runx2 is essential for terminal differentiation of peripheral pDCs. Runx2 was required for the expression of several pDC-enriched genes including the chemokine receptors Ccr2 and Ccr5 (Sawai et al., 2013). Finally, Stat5b and other members of the Stat family of transcriptional coactivators mediate GM-CSF-mediated stimulating effects on DC growth and terminal differentiation (Bontkes et al., 2006). In murine DCs and DC precursors, GM-CSF was shown to induce a unique set of the Stat family of signal transducers including Stat5b that in turn form heterodimeric signaling complexes to mediate GM-CSF-dependent signaling cascades (Welte et al., 1997). Stat5b supports diversification from CDPs toward cDCs by inhibiting pDC development. The Stat3/Stat5 signaling complex stimulates GM-CSF-mediated suppression of pDC-specific transcription factor E2-2 and supports induction of Id2 (Li et al., 2012). In addition, Stat5b suppresses GM-CSF-dependent generation of pDCs (Gilliet et al., 2002) through inhibition of Irf8 (Esashi et al., 2008). Stat5 also controls terminal differentiation of DCs at late developmental stages (van de Laar et al., 2011).

CDPs then give rise to pDCs and pre-DCs that are precursors of CD8^+^ and CD11b^+^ cDCs. In Flt3+ pDCs, expression of receptors for GM-CSF and M-CSF is down-regulated (Merad et al., 2013) since GM-CSF does not supports terminal differentiation of pDCs. However, M-CSF could drive development of both pDCs and cDCs from bone marrow-derived precursors in Fcl3-deficient mice suggesting that Flt3 is the archetypal DC poietin in the steady state (O'Keeffe et al., 2010). In Flt3-deficient mice, reduced numbers of MDPs and CDPs are remained. Indeed, Flt3 deficiency may restrict the ability of other growth factors to drive DC development by limiting the pool of progenitor cells available.

Type I IFNs (IFN-I) and Flt3 ligand could act synergistically to support pDC development (Chen et al., 2013). Flt3 ligand induces IFN-I expression in CLPs. IFN-I in turn activates Flt3 production in CLPs thereby enhancing CLP proliferation and differentiation to pDCs. On the surface, pDCs highly express IFN-α receptor (Ifnar) consisting of two chains (Ifnar1 and Ifnar2) and essential for binding IFN-I that are crucial for terminal maturation and steady-state function of pDCs (Gauzzi et al., 2002; Toma-Hirano et al., 2007).

Fully differentiated murine pDCs express a unique combination of surface markers including CD11c, B220, Ly6C/G, and Ly49Q (Omatsu et al., 2005). CD11c (or integrin αX) is expressed not only on pDCs but also on cDCs, monocytes, macrophages, and some B cells (Stewart et al., 1996). CD11c combines with the β 2 chain (ITGB2; CD18) to form a leukocyte-specific integrin referred to as inactivated-C3b (iC3b) receptor 4 (CR4; CD11c/CD18), which binds a variety of ligands that regulate pDC adhesion and motility (Piemonti et al., 1999). B220 is a surface receptor protein tyrosine phosphatase C (PtprcC or CD45) involved in the regulation of T- and B-cell antigen receptor signaling. In CD45-deficient mice, DCs had impaired capacity to produce IFN-I in response to lymphocytic choriomeningitis virus infection suggesting that CD45 is involved in the control of IFN-I production in pDCs (Montoya et al., 2006).

Expression of Ly49Q, a lectin-type killer cell inhibitory receptor, is induced by IFN-I (Toma-Hirano et al., 2007). On the other hand, Ly49Q is also involved in the regulation of IFN-I production by enhancing Toll-like receptor-9 (Tlr9)-mediated signaling in pDCs (Rahim et al., 2013). On pDCs, Ly49Q was shown to bind H-2K^b^, a classical MHC class I molecule (Tai et al., 2007), which enhances pDC function (Tai et al., 2008). Ly49Q also binds the Ly49Q ligand that stimulates maturation of pDCs (Toma-Hirano et al., 2009). Lack of Ly49Q expression defines two subsets of pDCs (Ly49Q^+^ and Ly49Q^−^ DCs) (Kamogawa-Schifter et al., 2005). Cultured Ly49Q^−^ cells are able to spontaneously up-regulate Ly49Q without cell proliferation and acquire most properties of typical pDCs. Upon stimulation with CpG oligonucleotides or Sendai virus, Ly49Q^−^ pDCs upregulate expression of CD86 and MHC class II but produce less IFN-I, IL-6, and IL-12 compared to Ly49Q^+^ pDCs (Omatsu et al., 2005). Therefore, Ly49Q^−^ cells represent less potent and less differentiated subset of pDCs.

Compared to CDPs, a variety of transcription factors and transcription regulators responsible for cell type-specific commitment is up-regulated in pDCs. Those include Bcl11a, Runx2, PU.1, E2-2, Spi-B, Irf4, Ifr8, and Stat3 (Seillet and Belz, 2013) (Table 1). In contrast, expression of Id2 and Nfil3 essential for diversification of common DC precursors is suppressed. In pDCs, expression of transcription factors such as Bcl6, Zbtb46, and Barf3 involved in cDC-specific differentiation is also down-regulated. E2-2 is highly expressed in human and mouse pDCs (Cisse et al., 2008). Deletion of E2-2 in mice results in lack of pDCs that suggests for specific role of this factor in the development of pDCs. E2-2 was found to be involved in activation of many pDC-enriched genes including transcription factors involved in pDC development (SpiB, Irf8) and function (Irf7) (Cisse et al., 2008). Spi-B, a PU.1-related transcription factor, is overexpressed in immediate pDC precursors and is able to support the development of myeloid lineage in PU.1-deficient mice (Dahl et al., 2002). However, Spi-B does not express in lymphoid cells (neuthrophils and monocytes) (Chen et al., 1995) because it prevents differentiation of hematopoietic progenitor cells toward the lymphoid lineage (Schotte et al., 2003). Spi-B is critical for development and function of both mouse (Sasaki et al., 2012) and human (Schotte et al., 2004) pDCs. E2-2 and Spi-B cooperate in stimulating pDC differentiation in order to overcome Id2 enforced block in pDC development (Nagasawa et al., 2008). In synergy with IRF7, Spi-B activates expression of IFN-I in pDCs (Sasaki et al., 2012). Finally, Spi-B supports survival of pDCs and their precursors by up-regulating expression of anti-apoptotic protein Bcl2-A1 (Karrich et al., 2012).

**Table 1:** **Expression of transcription regulators and receptors essential for differentiation of mouse DCs and their progenitors (precursors)**.

**Cell**	**Surface receptors**	**Transcription regulators**
GMP	Clec12a+++ C-kit++ Il3ra++	Stat5a+ Stat5b+
MDP	Clec12a++ C-kit++ Flt3+ Ccr2+ Cx3cr1+ Ifnar2+ Csf1r+	Nfil3+ Irf8+
CDP	Clec12a+ C-kit+ Flt3++ Ccr2+ Cx3cr1+ Ifnar2++ Csf1r+ Cd209+	Stat5b+ Nfil3++ Bcl11a++ Runx2++ Sfi1/PU.1++ Irf8+ Tcf4/2E-2+ Id2+ Zbtb46+ Spi-B+ Bcl6+
Pre-cDC	Clec12a+Cx3cr1+ Scf2ra++ Csf2rb+++ Csf2rb2+ Cxcl9++ Cxcl16+ Ifngr1++ Ly75+ Dxd58+ Tlr13+ Tlr3+	Stat5b+++ Stat3+ Bcl11a+++ Runx2+ Tcf4/2E-2+++ Spi-B+++ Irf8+++ Bcl6+ Irf4+++ Zbtb46+++
pDC	Clec12a+++ Tlr7+++ Tlr9+++ Flt3++ Csf2rb++ Csf2rb2+++ Ifnar1+++ Ifnar2+++ Il7r+++ Cx3cr1+++ Ccr9+++ Ccr2+ Siglech+++	Stat3++ Bcl11a+++ Runx2+++ Tcf4/2E-2+ Spi-B+++ Irf8+++ Irf4+++

*Data are adopted from Robbins et al. (2008) and Merad et al. (2013). The expression strength is indicated by the number of plus. Bcl, B-cell lymphoma; Ccr, C-C chemokine receptor; Cd209, cluster of differentiation 209, Dendritic Cell-Specific Intercellular adhesion molecule-3-Grabbing Non-integrin(GC-SIGN); C-kit, tyrosine-protein kinase Kit; Slec, C-type lectin domain; Csf1r, Colony stimulating factor 1 receptor; Cx3cr1, CX3C chemokine receptor 1; Cxcl, Chemokine (C-X-C motif) ligand; Dxd58, DEAD (Asp-Glu-Ala-Asp) box polypeptide 58; Id2, DNA-binding protein inhibitor 2; Flt3, Fms-like tyrosine kinase 3; Ifnar, interferon-α/β receptor; Ifngr1, interferon-γ receptor 1; Il3ra, interleukin-3 receptor, α-chain; Il7r, interleukin-7 receptor; Irf, Interferon regulatory factor; Ly75, Lymphocyte antigen 75; Nhil3, nuclear factor, interleukin-3 regulated 3; Runx2, Runt-related transcription factor 2; Siglech, Sialic acid-binding immunoglobulin-type lectin H; Stat, Signal transducer and activator of transcription; Sfi1, transcription factor PU.1; Spi-B, transcription factor (PU.1-related) Spi-B; Tcf4, transcription factor 4; Tlr, Toll-like receptor; Zbtb46, Zinc finger and BTB domain-containing protein 46*.

Compared to pre-DCs, Irf4 is significantly less up-regulated in pDCs (Robbins et al., 2008). Although Irf4 and Irf8 cooperatively act in supporting DC differentiation and share many overlapping activities (Yamamoto et al., 2011), Irf4 appears to preferentially support the development of CD4^+^CD11b^+^ cDCs while Irf8 is more focused on stimulating polarization of DC precursors toward pDC phenotype (Vander Lugt et al., 2014). Irf4 and Irf8 are differently regulated by GM-CSF, which suppresses Irf8 while promoting Irf4 expression (Esashi et al., 2008). In pre-DCs and cDCs, Irf4 target sequences are enriched with activating protein 1 (AP-1)-IRF composite elements (AICEs) capable to bind with Irf4/Batf3 complexes that further drive their transcription (Glasmacher et al., 2012). Compared to pre-DCs, Stat3/Stat5b ratio is skewed to Stat3 in pDCs since Stat3 supports terminal differentiation of CDP to pDCs whereas Stat5b expression promotes development toward cDCs (Cohen et al., 2008).

MDPs and CDPs generally produce large amounts cDCs and only few pDCs. Interestingly, Onai et al. (2013) reported identification of a Lin^−^c-kit^int/low^CD115^−^CD135^+^ progenitor with great capacity to differentiate predominantly to pDCs. In contrast to MDPs and CDPs in which expression of E2-2 is down-regulated, the progenitor expressed high levels of E2-2, an essential transcription factor for pDC development. The progenitors could be derived from either CDPs or lymphoid-primed multipotent progenitors (LMPPs) (Figure 1). Indeed, these findings could suggest for an alternative mechanism of the development of pDCs that should be further studied (Shortman and Sathe, 2013).

## pDC location and trafficking

DCs leave the bone marrow to give rise to resident and migratory DCs. In the lymphoid tissue, DCs present lymph- and blood-derived antigens to local T cells. Non-lymphoid tissue DCs permanently migrate from peripheral tissues to lymph nodes to present tissue-derived antigens to naïve T cells (Boltjes and van Wijk, 2014). Under homeostatic conditions, human and mouse pDCs are mainly resident cells. They are confined to primary and secondary lymphoid organs (lymph nodes, spleen). Unlike cDCs that reach the lymph node through afferent lymphatic vessels, pDCs and their precursors enter lymph nodes directly from the blood *via* endothelial venules (Segura et al., 2012). The migration of pDCs to lymphoid tissue is promoted by expression of L-selectin (non-inflamed states) or E-selectin (inflamed states) in high endothelial venules (Yoneyama et al., 2004). CCR7 highly expressed at the surface of pDCs triggers migration toward increased concentration gradient of its ligands CCL9 and CCL21 abundantly secreted by lymph nodes and then essentially contributes to maintaining pDC homing in lymph nodes (Seth et al., 2011). In the steady-state, resident pDCs express on the surface low levels of chemotactic molecules such as CXCR3, CXCR4, and chemokine receptor-like 1 (CMKLR1 or ChemR23) (Sozzani et al., 2010) that limits their motility.

In contrast to the current model of pDC development suggesting that mouse pDCs become fully differentiated in the bone marrow and then migrate to the periphery, Schlitzer et al. (2012) recently isolated a subset of circulating Ccr9^−^ pDC-like precursors from the mouse blood capable to differentiate either to pDCs or cDCs depending on the tissue. Adoptive transfer of these precursors to irradiated mice resulted in formation of Ccr9^+^ pDCs in the bone marrow and liver whereas in peripheral lymphoid organs, lung and intestine the precursors additionally gave rise to cDCs. The Ccr9- pDC-like precursors could be generated from CDP both *in vivo* and *in vitro* and able to migrate from the bone marrow to the peripheral lymphoid and non-lymphoid organs.

Compared to CDPs, the precursors expressed increased levels of pDC-lineage major transcription factors such as E2-2 and Sci-B and pDC-specific surface markers such as sialic acid-binding IgH-like lectin H (Siglec-H) and bone marrow stromal cell antigen Bst-2 (Blasius et al., 2006a,b) like terminally differentiated Ccr9+ pDCs. Interestingly, Ccr9− pDC-like precursors found by Schlitzer et al. (2012) share some functional and phenotypical properties with Lin^−^c-kit^int/low^CD115^−^CD135^+^ cells (Onai et al., 2013) including high potential to generate pDCs, elevated expression of E2-2, and capacity to be produced from CDPs but represent distinct pDC precursor pools. Indeed, pDCs precursors are likely to form a heterogeneous population. In contrast to Ccr9^+^ pDCs, Ccr9^−^ pDC-like precursors show flexibility to give rise other DCs subsets in a tissue-specific manner suggesting for the role of the local tissue microenvironment in determining the developmental fate of pDCs precursors in the peripheral organs. These local stimuli should be characterized in the future. The unique combination of soluble factors including M-CSF, Flt3 ligand, TPO, IL-3, and IFN-α (Chen et al., 2004; Demoulin et al., 2012) and unknown cell-bound factors is likely to contribute to making the final cell fate decision for differentiation into pDC or cDC subsets.

## Proinflammatory properties of fully differentiated pDCs

**Figure 2:**
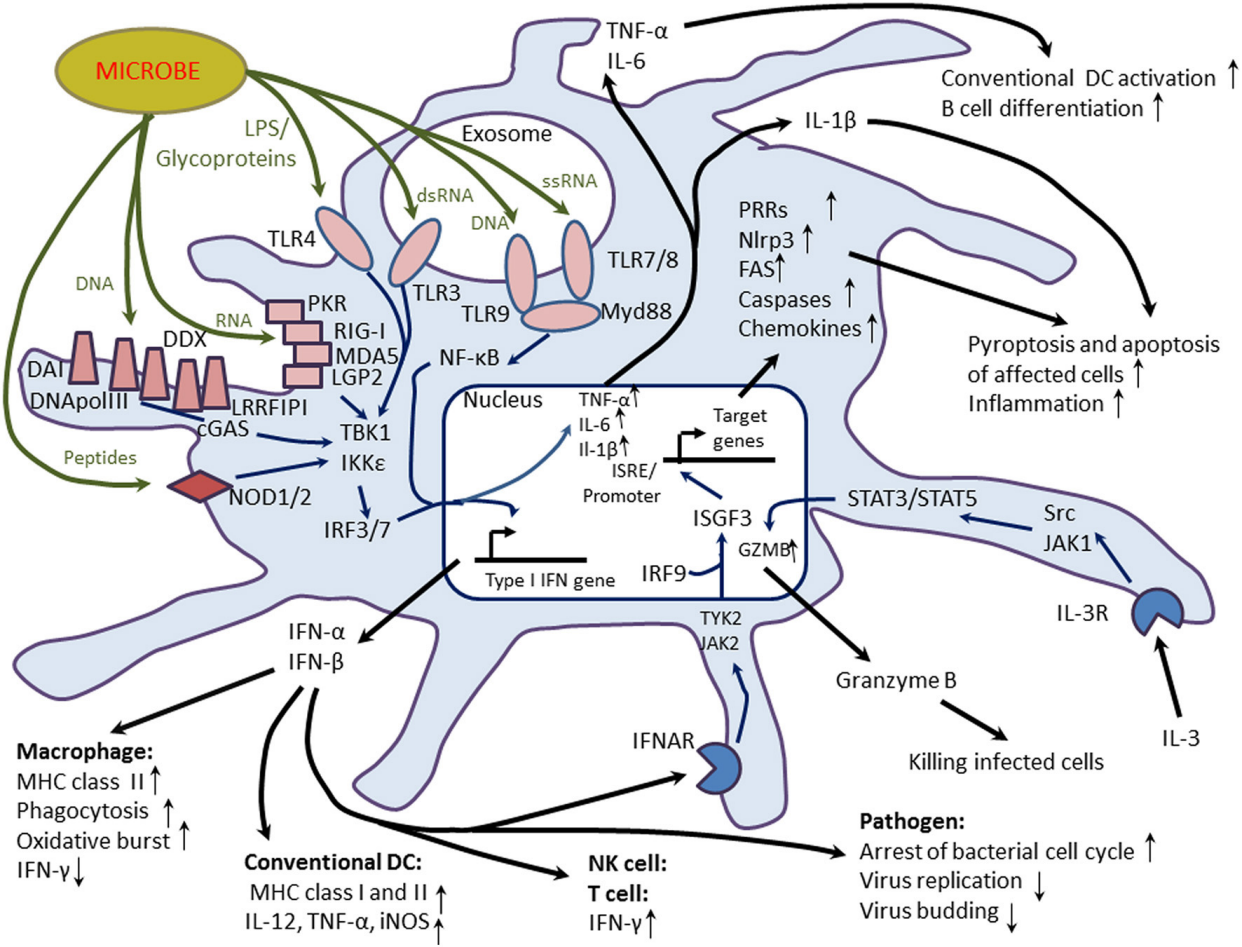
**Proinflammatory properties of plasmacytoid dendritic cells (pDCs)**. pDCs are capable to sense a variety of pathogen-associated molecular patterns (PAMPs) such as viral and bacterial peptides, peptidoglycans, lipopolysaccharides (LPS), RNA, and DNA through a range of pattern recognition receptors (PRRs) including Toll-like receptors (TLRs), double strand RNA-sensing receptor helicases (PRK, RIG-1, MDA5, and LGP2), and cytoplasmic receptors recognizing viral RNA and DNA (LRRFIPI, cGAS, DAI, DDX, and DNA polymerase III) or peptides (NOD1 and NOD2). TLR3, TLR7, TLR8, and TLR9 recognize pathogen-associated molecules in intracellular exosomes. PAMP-induced activation of PRRs leads to the activation of interferon-regulating factors IRF-3 and IRF-7 and transcription factor NF-κB that drive transcription of Type I interferon (IFN) genes such as IFN-α and IFN-β. In response to infection, pDCs secrete large amounts of IFN-α and IFN-β that display a variety of stimulatory activities on innate and adaptive immunity through activation of phagocytic and cytotoxic activities of macrophages, antigen-presenting function of conventional DCs (cDCs) and production of proinflammatory cytokines (interleukin(IL)-12, tumor necrosis factor (TNF)-α, and IFN-γ. IFN-I-induced up-regulation of IFN-γ production by natural killer (NK) cells and T cells is of great value since IFN-γ is a potent antiviral agent. IFN-I also inhibit virus replication and invasion and arrest bacterial proliferation. IFN-I is able to induce a positive feedback autocrine response through binding to the IFN-α/β receptor (IFNAR) on the surface of pDCs. The activation of the IFNAR leads to the recruitment of IRF9 that forms a heterotrimeric transcription complex IGGF3 (IFN-stimulated gene factor) capable to recognize specific regulatory motifs called interferon-stimulated response elements (ISRE) in the promoter regions of IFN-I-inducible target genes. As a result, many genes are expressed including PRRS, chemokines, caspases, inflammasome (cryopyrin, Nlrp3), and apoptotic cell surface death receptor Fas. Caspase-1 and caspase-11 are required for the activation of inflammasome components and IL-1β. Release of IL-1β and inflammasome induce death of infected cells through the pypoptotic mechanism and activates inflammatory response. pDCs also secrete IL-6 and TNF-α that activate cDCs and stimulate B cell differentiation to antibody-producing cells. pDCs could display cytotoxic properties in an IL-3-dependent manner. IL-3 produced by activated T cells could activate signaling pathway mediated by signal transducers and activator of transcription (STAT)3 and STAT5 and induce expression of granzyme B (GZMB), whose release causes death of infected host cells.

pDCs represents a specialized subset of DCs that phenotypically and functionally differs from cDCs. The primary function of pDCs is the recognition of pathogen-associated molecular patterns (PAMPs) such as viral single-strand RNA (through TLR7) or bacterial CpG nucleotide DNA sequences (through TLR9) and secretion of large amounts of IFN-I, IL-6, and TNF-α in response to infection (Rogers et al., 2013) (Figure 2). However, pDCs are also involved in the variety of other functions including supporting T cell survival, B cell differentiation (Shaw et al., 2010), cDC activation, and coordinating effector T cell-mediated immune responses against chronic infections (Cervantes-Barragan et al., 2012). On the surface, human and mouse pDCs express a variety of markers and receptors, which distinguish them from cDCs (Table 2). These molecules are involved in regulating key pDC functions such as antigen sensing and presentation, production of IFN-I, maturation, migration, adhesion and lineage maintenance.

**Table 2:** **Surface markers and receptors that distinguish human and mouse pDCs from cDCs**.

**Human pDC markers/receptors**	**Function**	**Mouse pDC markers/receptors**	**Function**
IL3Rα/CD123	Receptor for IL-3; supports terminal differentiation, maturation, and maintenance of pDCs	B220/CD45/PtprcC	Negative regulator of cytokine receptor signaling; regulation IFN-I production by pDCs
BDCA2/CLEC4A/CD303	Suppressing of TLR-9 mediated presentation of bacterial antigens; inhibition of IFN-I production	Siglec-H	Delivery of viral antigens to pDCs; regulation of IFN-I secretion through DAP-12
ILT7/LILRA4	Inhibition of production of IFN-I and proinflammatory cytokines; binds BST-2 (CD317)	Bst-2/CD317/mPDCA-1	Viral antigen delivery; inhibition of virus release from infected cells; interacts with the ligand, ILT7/LILRA4
ILT3/LILRB4	Antigen binding and presentation; binds MHC class I and inhibits activation of pDCs in response to antigen recognition	LyQ49	Stimulation of pDC maturation and function; activation of IFN-I production through TLR9-dependent signaling; binds LyQ49 ligand and MHC class I
LAIR1/CD305	Inhibition of pDC differentiation and function including IFN-I production; binds complement component c1q	Ly6C	Transmission of signals for T cell activation and cytokine production
BDCA4/NRP1	Regulation of cell adhesion and migration; binds semaphorins and VEGF	PDC-TREM	Regulation IFN-I production through DAP-12; interacts with plexin-A1
FcγRIIa/CD32	Phagocytosis of opsonized bacteria	Ly75/DEC205/CD205	Antigen delivery and presentation; recognition of apoptotic and necrotic cells
CD36	Antigen internalization for presentation to T cells	Lag-3/CD223	Regulation of pDC homeostasis; down-regulation of T cell activation; binds MHC class II
CD62L/L-selectin/SELL	Antigen internalization for presentation to T cells; attraction of pDCs and pDC precursors to lymph nodes; homing of pDCs in lymph nodes	CD200R	Induction of IDO production
IL-18R	Receptor for IL-18; recruits pDCs in response to inflammation	CCR9/CDw199	Regulation of homing of tolerogenic pDCs to the gut; binds CCL25
CCR7	Control of pDC maturation; attraction of pDCs to lymph nodes; homing of pDCs in lymph nodes; binds CCL19 and CCL21	IL7Rα/CD132	Receptor for IL-7; regulates maintenance of pDCs
TLR7	Recognition of viral single-strand RNAs; induction of IFN-I production	TLR7	Recognition of viral single-strand RNAs; induction of IFN-I production
TLR9	Recognition of bacterial unmethylated CpG sequences; induction of IFN-I and IL-12 production	TLR9	Recognition of bacterial unmethylated CpG sequences; induction of IFN-I and IL-12 production
CCR5	Recruitment of pDCs in response to inflammation; binds CCL3, CCL4, CCL5, and CCL3L1		
CXCR3	Recruitment of pDCs in response to viral infection and IFN-γ-mediated immunity; binds CXCL4, CXCL9, CXCL10, and CXCL11		

*BDCA2, Blood dendritic cell antigen 2; Bst-2, Bone marrow stromal cell antigen 2; DAP-12, DNAX-activating protein 12; DEC205, dendritic and epithelial cells, 205 kDa; FcγRIIa, Low affinity immunoglobulin-γ Fc region receptor IIa; IDO, indoleamine 2,3-dioxygenase; ILT7, Immunoglobulin-like transcript-7; Lag-3, Lymphocyte-activation gene 3; LAIR1, Leukocyte-associated immunoglobulin-like receptor 1; LILRA4, Leukocyte immunoglobulin-like receptor A4; MHC, Major histocompatibility complex; mPDCA-1, mouse plasmacytoid dendritic cell antigen-1; NRP1, Neurophilin 1; PDC- SELL, L-selectin; TREM, Plasmacytoid dendritic cell-specific receptor; PtprcC, Protein tyrosine phosphatase, receptor type C; VEGF, vascular endothelial growth factor*.

Mouse pDCs located in the spleen and liver express low levels of TLR4 capable to sense muramyl dipeptide (MDP) and other bacterial peptidoglycans and lipopolysaccharides (Uehori et al., 2005). MDP recognition is accompanied with increased expression of Nod2 (nucleotide-binding oligomerization domain-containing protein 2; also known as Card15 or Ibd1), the intracellular PAMP-recognizing receptor capable to sense peptidoglycans (Kufer et al., 2006). TLR4 was shown to be able detect not only bacterial pathogens but also recognize RNA viruses and lead to increased production of IFN-I, autophagy, and restricted virus replication (Kapoor et al., 2014).

In injury, the immune function of pDCs regulated by damage-associated molecular patterns (DAMPs) including self-DNA (recognized by TLR4 and TLR9) and high mobility group box 1 (HMGB1) recognized by receptor for advanced glycation end-products (RAGE) (Dumitriu et al., 2005; Bianchi, 2007). HMGB1 down-regulates TLR9-dependent capacity of pDCs to sense viral and bacterial antigens and stimulate proinflammatory immune responses mediated by T helper (Th)1 cells (Popovic et al., 2006). The inhibitory function of HMGB1 could suggest for the activation of protective tolerogenic mechanism focused on maintaining unresponsiveness of pDCs to self-antigens in the presence of necrotic cell death.

IFNs-I comprise a growing family of IFN proteins including IFN-α, IFN-β, IFN-δ, IFN-ε, IFN-κ, IFN-τ, IFN-ω, and IFN-ζ (Hertzog and Williams, 2013). Among multiple members of the IFN-I family, pDCs largely produce IFN-α and IFN-β, which bind to the common IFN-α/β receptor consisted of two chains (IFNAR1 and IFNAR2) (Uzé et al., 2007). Both components of the IFN-α/β receptor start to express in CDPs (Robbins et al., 2008). IFN-α supports terminal maturation and functional commitment of pDCs *via* binding to the IFN-α/β receptor (Luft et al., 1998; Korthals et al., 2007). IFN-α-dependent activation of the receptor recruits non-receptor tyrosine-protein kinases Tyk2 and Jak1 that phosphorylate several STAT members. STAT1 and STAT2 complex to the heterodimer that binds to IRF9 resulting in the formation of the heterotrimeric IFN-stimulated gene factor 3 (ISGF3) (Pestka et al., 2004) (Figure 2). The transcription complex translocates to the nucleus where it primes expression of many IFN-I-inducible gene targets including innate immune components such as TLR3, TLR7, TLR9, IRF7, double strand viral RNA-sensing helicases (RIG-1, MDA-5, and LGP-2), mitochondrial antiviral signaling protein (MAVS), and stimulator of interferon gene (STING) (Platanias, 2005). IFN-α also induces expression of inflammasome-forming components such as NOD-like receptor family, pyrin domain-containing 3 (NLRP3), retinoic acid-inducible gene 1 (RIG-I), and CD69 (Malireddi and Kanneganti, 2013) and up-regulates caspase-11 essential for activation of inflammasome (Case et al., 2013).

IFN-I possess strong inflammatory properties by activating the non-canonical NLRP3 inflammasome and contributing to the inflammasome-dependent caspase-1 activation that leads to proinflammatory pyroptotic cell death (Anand et al., 2011). Pyropoptosis is associated with production of IL-1β and IL-18 that increases the proinflammatory microenvironment in infected and wounded tissues (Anand et al., 2011). pDCs also contribute to inflammation by performing granzyme B- and caspase-dependent cytotoxicity against target cells. The cytotoxic properties of activated pDCs are up-regulated by IL-3 under inflammatory conditions (Bratke et al., 2010). Finally, stimulated pDCs produce increased levels of chemokines (CCL3, CCL4, CCL5, CXCL9, and CXCL10) that attract inflammatory cells to the inflamed sites (Sozzani et al., 2010).

## Tolerogenic properties of pDCs

**Figure 3:**
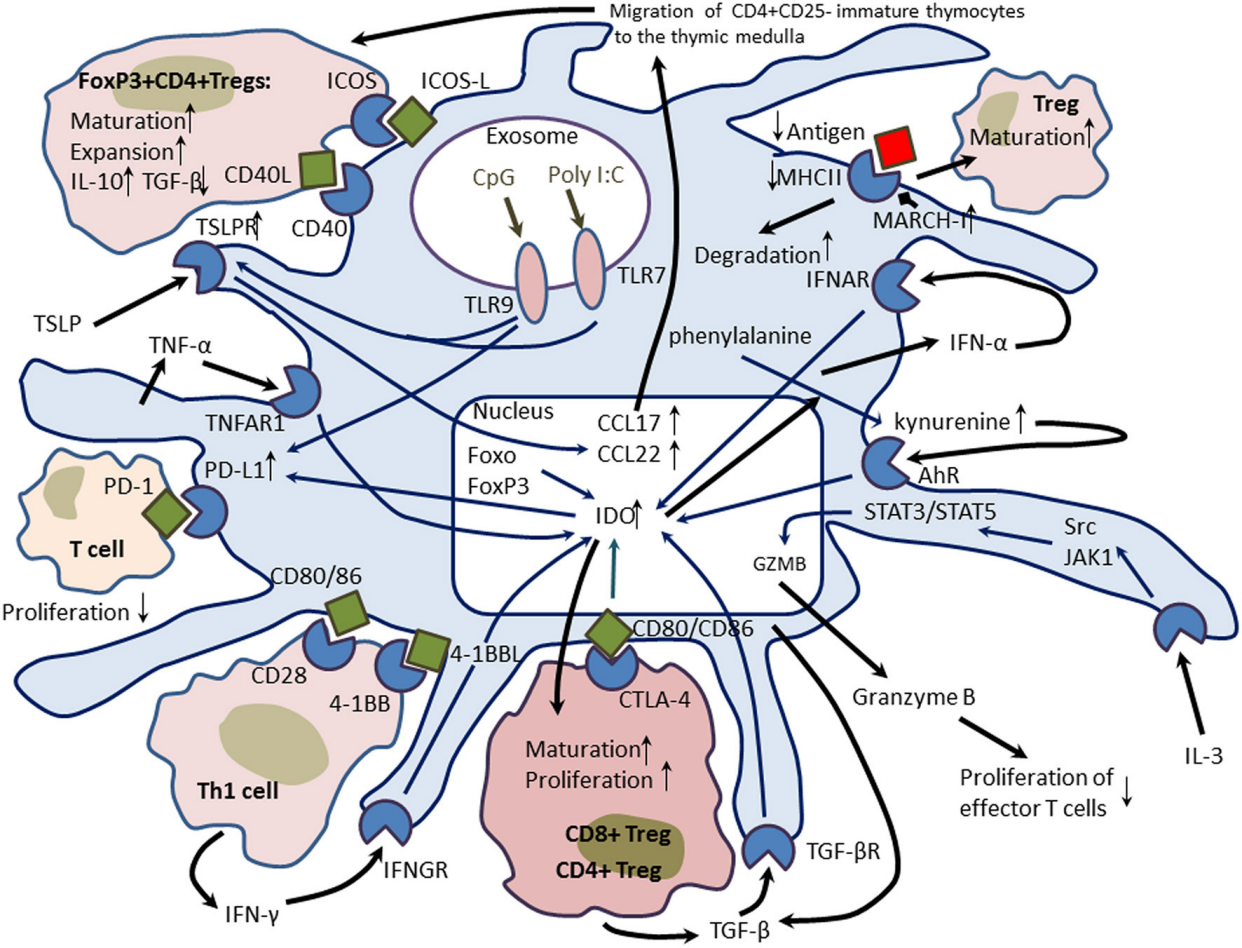
**Tolerogenic properties of plasmacytoid dendritic cells (pDCs)**. pDCs utilize several molecular mechanisms to induce tolerogenic properties. When phosphorylated, indoleamine-pyrrole 2,3-dioxygenase (IDO), an enzyme catabolizing L-tryptophan to N-formylkynurenine acquires potent immunomodulatory properties. IDO promotes differentiation of naïve T cells to inducible CD4^+^ and CD8^+^ regulatory T cells (Tregs) through cytotoxic T-lymphocyte antigen 4 (CTLA4)/CD80 pathway. In pDCs, IDO expression could be induced by multiple mechanisms including interferon (IFN)-γ-, IFN-α-, and transforming growth factor (TNF)-β-dependent pathways. Transcription factors Foxo and FoxP3 also stimulate IDO production. Kynurenine, a product of the IDO-catalyzed reaction can up-regulate IDO via a positive autocrine feedback mediated by the aryl-hydrocarbon receptor (AhR). IDO can up-regulate expression of the immunosuppressive molecule PD-L1 (programmed death-ligand 1) on the surface of pDCs. PD-L1 interaction with the PD-1 molecule decreases proliferation of the target T cell. Stimulation of the Toll-like receptor (TLR)-9 with CpG oligonucleotides leads to the activation of PD-L1 and induces expression thymic stromal lymphopoietin (TSLP) receptor on the surface of pDCs. The TSLPR is essential to mediate stimulatory effects of TSLP produced by Hassall's corpuscles on pDC-mediated differentiation, maturation, and expansion of immature thymocytes to CD4^+^CD25^+^FoxP3^+^ Tregs and promoting central tolerance. TSLP-activated pDCs produce chemokines CCl17 and CCL21 essential for attraction of thymocytes to the thymic medulla. TSLP-induced pDCs drive maturation of Tregs that produce more IL-10 and less TGF-β. On the surface, pDCs express unstable antigen-Major Histocompatibility Complex (MHC) class II due to up-regulated membrane-associated MHC II ubiquitin E3 ligase MARCH-I that promotes rapid turnover and degradation of antigen-presenting complexes and supports maturation of Tregs. Finally, IL-3 could activate expression of granzyme B in pDCs, and its release suppresses proliferation of effector T cells.

On the other hand, pDCs were shown to display prominent tolerogenic and immunosuppressive activities (Figure 3). Compared to cDCs, pDCs possess poor properties to stimulate T cells because they have a limited capacity to endocytosis and express low surface levels of MHC class II, costimulatory molecules, and cathepsins S and D essential for antigen processing (Rogers et al., 2013). pDCs were found in the cortical and medullar layers of the thymus where they play a role in inducing and maintaining central tolerance. In mice, thymus-associated tolerogenic pDCs are mainly of extrathymic origin. Like cDCs, thymic pDCs acquire advanced ability to endocytose, process, and present peripheral antigens to central tolerance. The antigen-presenting capacity is enhanced through increased surface expression of CD8a, CD11c, and MHC class II (Hadeiba et al., 2012). CCR9 and absence of TLR stimulation drives thymic migration of pDCs and further homing in the thymus. CCR9^+^ pDCs are potent inducers of regulatory T cells (Tregs) that are capable to suppress antigen-specific immune responses both *in vitro* and *in vivo* (Hadeiba et al., 2008). However, compared to Sirpα^+^ cDCs and CD8α^+^ cDCs, pDCs were shown to be less efficient in the presentation of blood-borne antigens followed with negative selection of T cells and induction of Tregs (Atibalentja et al., 2011). Martín et al. (2002) characterized population of mouse thymic CD8α^+^B220^+^ pDCs with regulatory properties capable to induce Tregs that further suppressed antigen-specific T-cell proliferation. Upon stimulation with artificial microbial antigens (CpG oligonucleotides, poly I:C), these cells were able to differentiate to potent APCs producing IFN-I, IL-10, and IL-12 (Martín et al., 2002).

In humans, thymic resident pDCs were shown to induce natural FoxP3^+^CD4^+^CD25^+^ Tregs (nTregs) from thymic precursors in a CD40/CD40L-dependent manner (Martín-Gayo et al., 2010). pDC-induced nTregs preferentially produced anti-inflammatory cytokine IL-10 while cDC-induced nTregs were better TGF-β producers. Indeed, the CD40/CD40L-dependent signaling may play a central role in induction of tolerogenic immunoregulatory pDCs in both humans and mice (Kumanogoh et al., 2001). nTreg precursors stimulate CD40L on T-cell receptor (TCR) engagement and induce maturation of pDCs. Therefore, CD40-CD40L-mediated crosstalk between pDCs and self-reactive nTreg precursors may provide critical feedback signals required for pDC maturation and nTreg differentiation in the steady-state human thymus (Martín-Gayo et al., 2010).

Thymus-specific structures formed by epithelial reticular cells in human and murine thymic medulla called Hassall's corpuscles were shown to secrete thymic stromal lymphopoietin (TSLP), a hemapoietic growth factor that is involved in pDC-induced generation of nTregs (Watanabe et al., 2005). TSLP up-regulates CD80/CD86 in thymic CD11c+ DCs that became able to drive secondary positive selection of self-reactive CD4^+^CD8^−^CD25^−^ T cells to forkhead box P3 (FoxP3)^+^ nTregs. The TSLP-dependent mechanism of generating nTregs involves TLR7- and TLR9-mediated induction of the TSLP receptor and IL-7Rα in pDCs that start to secrete chemokines CCL17 and CCL22 guiding the traffic of developing immature thymic T cells into the medulla (Hanabuchi et al., 2010). TLR9 stimulation with CpG oligonucleotides is shown to play a key role in pDC-mediated induction of both thymic and peripheral FoxP3+CD4+CD25+ Tregs (Moseman et al., 2004). Interestingly, TSLP-induced pDCs support generation of IL-10-producing nTregs while TSLP-induced cDCs contribute to preferential differentiation of TGF-β-producing Tregs. The two subsets of nTregs could be distinguished not only by cytokine production but presence/absence of inducible T-cell costimulator (ICOS). The ICOS^+^ nTregs subset had the potential to express high IL-10 and less TGF-β (Ito et al., 2008). Therefore, thymic pDCs and cDCs are able to induce distinct subsets of nTregs.

In the periphery, antigen-stimulated pDCs were found to support differentiation of naïve CD4^+^ T cells to effector T cells producing high levels of anti-inflammatory IL-10 and IFN-γ (Kvale et al., 2007) or to cytotoxic CD4^+^ T cells with regulatory properties (Kawamura et al., 2006) in order to prevent unfavorable inflammatory response and induce mucosal tolerance to ingested (gut) or inhaled (airways) antigens. Murine liver pDCs induce antigen-specific suppression of CD4^+^CD8^+^ T cell responses mediated by deletion or anergy (Mann et al., 2013). Peripheral pDCs also induce various tissue-specific Treg subsets (for example, such as tonsilar CD4^+^CD25^+^CD127^−^FoxP3^+^ Tregs (Palomares et al., 2012) from naïve T cells responsible for maintaining peripheral tolerance. pDC also imprint specific homing properties on T cells that they stimulate enabling T-cell migration back to home sites (Lombardi and Khaiboullina, 2014).

pDCs utilize several immunosuppressive tolerogenic molecular mechanisms including induction of indoleamine-pyrrole 2,3-dioxygenase (IDO), an enzyme catabolizing L-tryptophan to N-formylkynurenine (Fallarino et al., 2007; Kahler and Mellor, 2009). This enzyme possesses immunomodulatory (immunosuppressive) properties by removing the essential amino acid tryptophan from the cellular microenvironment (Mellor, 2005). In pDCs, IDO could be induced through non-canonical NF-κ B signaling by CD200-Ig-dependent stimulation of CD200 receptor (CD200R) with the involvement of the IFN-α/β receptor signaling (Fallarino et al., 2004), IFN-γ-mediated feedback (with induction of the IDO enzymatic function), and in a TGF-β-dependent manner (with induction of the IDO regulatory function) (Fallarino et al., 2012). IFN-γ was shown to induce the IRF-8-mediated expression of IDO associated with down-regulation of DAP-12, a negative regulator of the IDO production (Orabona et al., 2005, 2006). Once phosphorylated, IDO mediates signaling events culminating in self-amplification and maintenance of a stably regulatory condition in pDCs (Heitger, 2011). IDO promotes differentiation of naïve CD4^+^ T cells to Tregs by stimulating CD40/CD40L-mediated signaling through cytotoxic T-lymphocyte antigen 4 (CTLA4)/CD80, CD40/CD40L, and glucocorticoid-induced tumor necrosis factor receptor (GITR)/CITR ligand mechanisms (Fallarino et al., 2005). A positive feedback loop mediated by kynurenine through the aryl-hydrocarbon receptor (AhR) on the surface of pDCs and stimulatory signals from Tregs are required for maintenance of IDO-dependent tolerogenic properties of pDCs (Harden and Egilmez, 2012).

By down-regulating membrane-associated MHC II ubiquitin E3 ligase MARCH-I, cDCs were shown to form stable complexes between antigenic and MHC class II molecules on their surface upon activation (De Gassart et al., 2008; Ohmura-Hoshino et al., 2009) that supports the long-term antigen-presenting function. In contrast, activated pDCs failed to inhibit MARCH-I that results in formation of continuously ubiquitinated, internalized, and unstable antigen-MHC class II complexes on their surface (Young et al., 2008). Since low antigen levels (and low TCR stimuli) were found to promote development of Tregs (Turner et al., 2009; Molinero et al., 2011), the rapid turnover of antigen-MHC class complexes on the surface of pDCs underlines their limited antigen-presenting ability and therefore supports low antigen levels, which in turn should stimulate MARCH-I-mediated induction and functional activity of Tregs (Ishido et al., 2010).

Another pDC-mediated immunosuppressive mechanism involves up-regulation of the surface expression of programmed death-ligand 1 (PD-L1), an immunoinhibitory molecule (Keir et al., 2007). Interaction between PD-1 and its ligand PD-L1 promotes tolerance by blocking the TCR-induced stop signal in the target T cell (Fife et al., 2009). IL-27 was shown to initiate PD-L1 expression in pDCs in a STAT3-dependent manner thereby inducing their immunosuppressive properties and limiting the potential to stimulate naïve T cells (Karakhanova et al., 2011). Mouse liver pDCs were shown to express high levels of IL-27 and IL-27 receptor, which are essential for induction of PD-L1-dependent tolerogenic properties of pDCs (Matta et al., 2012).

Indeed, these data clearly show that pDCs could display proinflammatory and immunosuppressive tolerogenic properties. The local microenvironment and extrinsic stimuli influence pDC phenotype and hence could control the phenotypic switch toward inflammation or tolerance. pDCs were shown to be involved in advanced inflammatory response in several autoimmune proinflammatory diseases including multiple sclerosis, inflammatory bowel disease, psoriasis, and systemic lupus erythematosus (von Glehn et al., 2012). On the other hand, pDCs drive development of Tregs and mediate immunosuppression and tolerance in graft-versus-host disease (Rogers et al., 2013) and some cancers (Johnson and Ohashi, 2013). Atherogenesis was shown to involve many types of immune cells that display a variety of activities in supporting or suppressing atherosclerosis-associated inflammation. Among those, pDCs play a non-redundant role in atherogenesis (Döring and Zernecke, 2012; Alberts-Grill et al., 2013; Ait-Oufella et al., 2014; Busch et al., 2014; Subramanian and Tabas, 2014; Zernecke, 2014).

## Proinflammatory activities of pDCs in atherosclerosis

**Figure 4:**
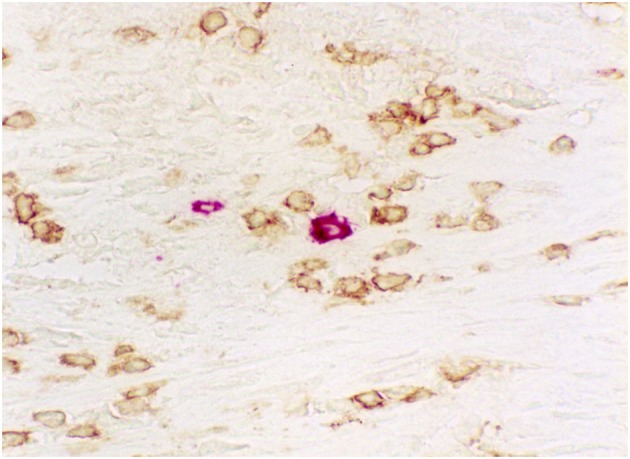
**Immunohistochemical visualization of close proximity of pDCs (rose) to T cells (brown) in an area of human aortic atherosclerotic plaque containing immune-inflammatory infiltrate**. Double immunostaining: pDCs were visualized using anti- BDCA-2 antibody (CD303; Miltenyi Biotec) while T cells were visualized using anti-CD3 antibody (Dako). A combination of peroxidase-antiperoxidase and alkaline phosphatase-antialkaline phosphatase immunotechniques using 3-Amino-9-ethylcarbazole (AEC) substrate and diaminobenzidine (DAB). Magnification: ×400.

pDCs were found to be colocalized with T cells in atherosclerotic plaques in humans (Yilmaz et al., 2004; Bobryshev, 2010) and mice (Jongstra-Bilen et al., 2006) preferentially in the unstable shoulder region (Yilmaz et al., 2004; Niessner et al., 2006). Close proximity of pDCs to T cells in plaque areas, containing immune-inflammatory infiltrates, is indicative that the functional contacts between pDCs and T cells might be formed directly *in situ* in atherosclerotic arteries (Figure 4).

There are some conflicting results regarding the changes in pDC numbers during atherosclerosis progression. Some studies reported unchanged counts of pDCs in patients with coronary artery disease (CAD) (Shi et al., 2007) and myocardial infarction (MI) (Wen et al., 2013). Other researchers detected decreased numbers of pDCs in patients with troponin-positive unstable coronary artery syndromes (Van Vré et al., 2006), end-stage heart disease (Athanassopoulos et al., 2004), and advanced CAD (Yilmaz et al., 2009; Van Vré et al., 2010). Furthermore, an inverse correlation between the severity of CAD and pDC numbers was observed (Yilmaz et al., 2009; Van Brussel et al., 2011). Decrease in pDC levels observed in acute coronary events could reflect increased recruitment of pDCs in supporting advanced pronflammatory responses preceding the acute cardiovascular event (Sorrentino et al., 2010). There were no significant changes in numbers of circulating pDC precursors in CAD patients (Wen et al., 2013) although concentration of pDC attractants such as CCL2, CCL5, CXCL12, E-selectin, and P-selectin is increased in atherosclerotic vessels (Peter et al., 1997; de La Rosa et al., 2003; Erbel et al., 2007).

In atherosclerosis, pDC function is impaired. Plaque tissues of patients with ischemic complications (and shoulder regions especially) expressed higher levels of CD83 (Siglec), a marker of DC activation (Erbel et al., 2007). However, Van Brussel et al. (2011) showed that pDCs derived from CAD patients expressed lower levels of CD83 and TLR7 and as a consequence produced less IFN-α. Similarly, decreased expression of proinflammatory cytokines (IL-1β and IL-6) was observed in type 2 diabetic patients with atherosclerotic complications (Corrales et al., 2007).This may suggest for impaired function of atherosclerotic pDCs that become more resistant to multiple proinflammatory stimuli present in the plaque. Otherwise, this may be a consequence of the negative autocrine regulatory feedback of IFN-α (Malireddi and Kanneganti, 2013).

Modified lipoproteins are considered to be one of the major inducers and mediators of atherosclerosis-related inflammation. Oxidized low density lipoproteins (oxLDL) were shown to prime differentiation and maturation of cultured pDC precursors to cDCs and pDCs *in vitro* (Nickel et al., 2009) suggesting for possibility of oxLDL-driven proatherosclerotic differentiation of pDC precursors in vascular inflammation. The oxLDL-dependent DC differentiation is mediated by scavenger receptors such as CD36 and CD205 whose expression is up-regulated. CD36 is a principal receptor that is involved in oxLDL uptake by DCs and macrophages (Kunjathoor et al., 2002). In atherosclerosis, pDCs could be also activated with a variety of antigens such as self-DNA, antimicrobial peptide Cramp/LL37 (Döring et al., 2012), and CpG oligonucleotides (Niessner et al., 2006). Self-antigens are able to form circulating immune complexes with self-antibodies that possess advanced atherogenic properties through stimulation of T-cell-mediated proinflammatory responses. Self-DNA can form complexes with LL37, which then activate IFN-α production by pDCs through the TCR9-mediated signaling (Lande et al., 2007).

Activated pDCs were shown to support atherosclerosis progression by elevated production of proinflammatory IFN-α (Niessner et al., 2006). In both mouse atherosclerosis models [e.g., animals deficient for apolipoprotein E (ApoE) or LDL receptor (Ldlr)], treatment with IFN-I enhanced atherogenesis associated with increase in lesion numbers and plaque size, macrophage-endothelial cell adhesion, and leukocyte attraction to inflamed vascular sites (Levy et al., 2003; Goossens et al., 2010). In ApoE-deficient mice, specific depletion of pDCs with mPDCA-1 antibody had the atheroprotective effect by reducing accumulation of macrophages in plaques and T-cell activation, decreasing production of proinflammatory cytokines (IL-12, IFN-γ) and chemokines (CXCL1, CXCL10), increasing plaque fibrosis, and implying a more stable lesion phenotype (Macritchie et al., 2012).

In humans, pDC-mediated secretion of IFN-α led to the proinflammatory activation of cDCs that is reflected by up-regulated TLR4-mediated production of a set of inflammatory cytokines (IL-12, IL-23, and TNF-α) (Niessner et al., 2007). In addition, IFN-α induced increased IFN-γ secretion and up-regulation of TNF-related apoptosis-inducing ligand (TRAIL) in effector T cells (Niessner et al., 2006). TRAIL-positive CD4^+^ T cells were shown to kill vascular smooth muscle cells (VSMCs) in the atherosclerotic plaque *via* the TRAIL/DR5-dependent apoptotic mechanism (Pryshchep et al., 2006; Sato et al., 2006). Indeed, pDC-induced activation of atherogenic cytotoxic T cells could cause VSMC apoptosis, a process that could lead to plaque destabilization and increase risk of acute coronary syndrome. Human pDCs were shown to possess cytotoxic properties by IL-3-dependent releasing granzyme B that kills endothelial cells (Bratke et al., 2010).

## Tolerogenic and immunosuppressive properties of pDCs in atherosclerosis

Recently, Daissormont et al. (2011) showed atheroprotective properties of pDCs in in Ldlr-deficient mice. Targeted depletion of pDCs with a Bst-2-specific antibody aggravated atherogenic progression associated with increased T cell accumulation in plaques and elevated IFN-γ production. The depleted pDC population showed tolerogenic and anti-inflammatory properties *via* the IDO-dependent mechanism since IDO inhibition disrupted the immunosuppressive effect of pDCs. In addition to IDO, tolerogenic pDCs could involve other immunosuppressive mechanisms such as PD1/PD-L1 (Tokita et al., 2008) and IL-3-mediated granzyme B production to suppress proliferation of effector T cells (Jahrsdörfer et al., 2010). It appears that only certain pDC subsets including immature and tissue-specific pDCs (Ochando et al., 2006; Hadeiba et al., 2008) or pDC precursors (MacDonald et al., 2005; Huang et al., 2011) could exhibit immunoregulatory properties and support induction of T regs. In murine atherosclerotic plaques, tolerogenic pDCs are scarcely presented (Daissormont et al. (2011). However, they are able to influence significantly the atherosclerotic progression.

Interestingly, atheroprotective tolerogenic pDCs could be induced by self-peptides such as an ApoE-derived peptide Ep1.B (Bellemore et al., 2014) or with bacterial antigens such as *Mycobacterium bovis* BCG (a causative agent of tuberculosis) killed by extended freeze-drying (BCG EFD) (Ovchinnikova et al., 2014). The ApoE peptide Ep1.B corresponds to C-terminal amino acids 239-252 of the human ApoE molecule and is atheroperotective itself (Stephens et al., 2008). When injected to the mice, the peptide was shown to induce molecular and phenotypic changes in pDCs inducing tolerogenic properties associated with plaque degradation, diminished IFN-γ production and proliferation of T cells, activation of IL-10 secretion, and generation of Treg cells (Bocksch et al., 2007). Similar anti-atherosclerotic effects were reached with pDCs induced with Ep1.B and BCG EFD including induction of IL-10-producing Tregs (Ovchinnikova et al., 2014). Tolerogenic pDCs are likely to drive development of inducible Tregs in draining lymph nodes followed with expansion of Tregs but not tolerogenic pDCs in spleen.

These experiments provide a prominent promise for using tolerogenic pDCs for development of cost-effective atheroprotective vaccines based on available immunogenes such as BCG EFD. However, DC-based anti-atherosclerotic immunization protocols have been only recently employed and much more should be done in developing efficient vaccination strategies for atherosclerosis. One of the advances in developing effective vaccines for atherosclerosis is the selection of a specific antigen to target. The ApoE peptide Ep1.B could represent such an antigen that is beneficial for obtaining tolerogenic pDC-based atheroprotective vaccines.

## Conclusion

Thus, pDCs influence the development of atherosclerosis in both ways, by contributing to proatherogenic vascular inflammation and by suppressing inflammatory responses through induction of self-tolerogenic properties and Tregs. It is likely that pDCs preferentially exhibit the proinflammatory phenotype at early atherosclerosis stages. At initial steps of lesion formation, pDCs activated by proinflammatory stimuli produce proatherogenic IFN-I and trigger T cell activation and T cell-mediated immune responses including generation of inducible Tregs. In advanced atherosclerotic lesions, pDCs could acquire tolerogenic properties and delay the development of atherosclerosis. However, to verify this hypothesis, sequential observations of quantitative and qualitative changes in the function and phenotype of pDCs during atherosclerosis progression are required.

### Conflict of interest statement

The authors declare that the research was conducted in the absence of any commercial or financial relationships that could be construed as a potential conflict of interest.
